# Rapid Diagnostic Model for Critical Illness Polyneuropathy Based on Electrophysiological Data

**DOI:** 10.1111/cns.70631

**Published:** 2025-10-22

**Authors:** Yang Liu, Zihan Zhang, Zihan Jing, Binbin Sun, Hong Zhao, Yan Wang, Mao Li, Dehao Li, Dengfa Zhao, Hongmei Cheng, Min Su, Haoyang Hu, Ruozhuo Liu, Shengyuan Yu, Fei Yang

**Affiliations:** ^1^ Department of Neurology, The First Medical Center Chinese PLA General Hospital Beijing China; ^2^ Neurology Institute of Chinese PLA General Hospital Beijing China; ^3^ School of Medicine Nankai University Tianjin China; ^4^ Department of Neurology Nanfang Hospital Guangzhou China; ^5^ Department of Neurology, The Fifth Medical Center Chinese PLA General Hospital Beijing China

**Keywords:** critical illness polyneuropathy, electrophysiological data, intensive care unit, machine learning, rapid diagnostic model

## Abstract

**Background:**

Critical illness polyneuropathy (CIP) is a common cause of weakness in critically ill patients, and early diagnosis and treatment are essential. Conventional comprehensive electrophysiological testing requires 60–90 min and can be invasive, limiting its utility in the intensive care setting. This study aimed to develop a rapid, efficient, and minimally invasive diagnostic model for CIP.

**Methods:**

Patients who met the inclusion criteria were recruited from the intensive care units (ICU) of the First Medical Center and Fifth Medical Center of the PLA General Hospital and Nanfang Hospital. To identify the optimal diagnostic model, we compared several machine learning approaches, including K‐nearest neighbor, support vector machine with radial basis function (SVM‐RBF), SVM with Gaussian kernel, random forest, and extreme gradient boosting (XGB), using all electrophysiological features. These were also compared with nerve conduction studies of the peroneal and sural nerves. To construct a rapid diagnostic model, different feature combinations were assessed across the machine learning methods. Model performance was evaluated using cross‐validated areas under the receiver operating characteristic curve (AUCs).

**Results:**

Of 14,768 admissions screened, 134 patients with CIP and 135 matched controls were included. In total, 41 electrophysiological features were analyzed. Feature ranking revealed that the distal compound muscle action potential (CMAP) of the peroneal nerve contributed most to diagnostic accuracy. After comparison, the SVM‐RBF method was selected to establish the final diagnostic model. The optimal model, based on seven electrophysiological features of the peroneal and ulnar nerves (proximal latency, distal latency, and CMAP amplitude), achieved an AUC of 0.93, which was comparable to the all‐feature XGB model (AUC = 0.95). In the independent validation set, the rapid diagnostic model maintained strong performance (AUC = 0.88), similar to the all‐feature model (AUC = 0.90).

**Conclusion:**

We developed a rapid diagnostic model for CIP using the SVM‐RBF method and seven electrophysiological features from the peroneal and ulnar nerves. This model enables efficient, minimally invasive, and timely diagnosis of CIP in critically ill patients. The source code and related scripts are available at [https://github.com/PLAGH‐Neuro‐Yang/RDM‐CIP].

## Graphical abstract

A rapid diagnostic model was developed for CIP in ICU patients using seven electrophysiological indices from the peroneal and ulnar nerves with the SVM approach. This model demonstrated superior diagnostic accuracy compared with the conventional method. It reduced diagnostic time to 10–20 min, representing a substantial improvement over conventional testing. These results support the clinical utility of this model and highlight its potential to facilitate timely diagnosis of patients with CIP.

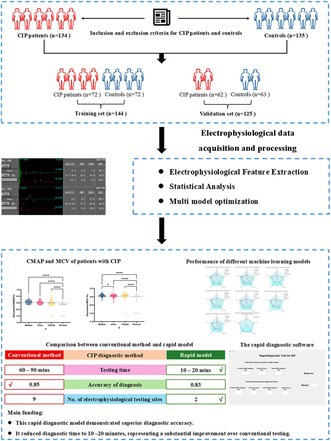

## 1 Introduction

Intensive care unit‐acquired weakness (ICUAW) is defined as new‐onset weakness in critically ill patients that develops during the course of intensive care unit (ICU) admission, in the absence of other etiologies beyond critical illness itself [1, 2]. ICUAW may result from critical illness polyneuropathy (CIP), critical illness myopathy (CIM), or critical illness polyneuropathy (CIPNM) [3, 4]. Differentiation between these subtypes depends on clinical, electrophysiological assessments, and histological evidence [5]. Among them, CIP is particularly concerning due to its association with higher mortality rates and persistent frailty following hospital discharge. In contrast, CIM and CIPNM are more readily detected through elevated muscle enzymes and overt muscular weakness. However, CIP lacks specific laboratory biomarkers, and associated sensory abnormalities are often overlooked [6, 7]. CIP can be distinguished from deconditioning states, which present as generalized weakness without electrophysiological abnormalities and typically carry a better prognosis [4, 8, 9, 10, 11]. Therefore, early diagnosis and timely treatment of CIP are critical, with electrophysiological testing remaining central to its diagnosis.

Despite this importance, conventional electrophysiological testing has notable limitations in diagnosing CIP. First, comprehensive testing is time‐consuming and labor‐intensive, typically requiring 60–90 min to complete in ICU patients [12]. Second, needle electromyography depends heavily on operator expertise and patient cooperation. While previous studies suggest that simplified approaches, such as assessing the distal compound muscle action potential (CMAP) of the peroneal nerve, can aid diagnosis [12, 13, 14, 15, 16], reliance on a single nerve measure may be confounded by conditions such as acute unilateral peroneal nerve palsy due to trauma, surgery, or other diseases [17, 18]. Consequently, depending solely on peroneal nerve testing may result in high false‐positive rates [19]. These challenges underscore the need for economical, efficient, and reliable diagnostic methods.

In the broader field of neurology, electrophysiological signals such as electromyography (EMG) and electroencephalography (EEG) are regarded as robust indicators of neuromuscular disorders and cognitive dysfunction. Recently, machine learning methods have increasingly been applied to develop diagnostic models in this domain. Commonly used approaches include K‐nearest neighbor (KNN), support vector machines (SVM), random forest (RF), and extreme gradient boosting (XGB), with method selection often dependent on task‐specific requirements and available resources. The success of these models relies on appropriate feature extraction, parameter optimization, and sufficient data size and recording quality [20].

In this study, we developed a rapid electrophysiological diagnostic model for CIP using the SVM method. This model has the potential to significantly improve diagnostic efficiency in critically ill patients.

## 2 Methods

### 2.1 Patients and Study Design

This study was approved by the Ethics Committee of the Chinese People's Liberation Army General Hospital (IEC No. S2022‐706‐01), and written informed consent was obtained from all the patients at the time of electrophysiological examination.

Clinical, laboratory, and electrophysiological data were collected from patients who met the inclusion criteria and were admitted to ICU (neurology, respiratory, surgical, cardiovascular ICUs) at the First Medical Center and Fifth Medical Center of PLA General Hospital, as well as Nanfang Hospital, between October 2018 and June 2022. For patients who underwent more than one laboratory or electrophysiological examination, results obtained at the time of the most severe symptoms were used for analysis.

Patients were included if they met the following criteria: presence of organ dysfunction leading to life‐threatening conditions, such as severe sepsis or septic shock, severe intracranial infections, severe cerebrovascular diseases (e.g., nontraumatic subarachnoid or intracerebral hemorrhage), or other critical organ failures such as acute respiratory failure [15, 21]; new onset of limb and/or generalized weakness during ICU admission or weaning that could not be explained by the primary disease, defined as a Medical Research Council (MRC) score ≤ 48 [22] or clinical weakness on physical examination [8, 23]; and electrophysiological findings consistent with CIP [24]. Electrophysiological evidence of CIP was defined as motor and sensory axonal polyneuropathy, with at least two CMAP amplitudes < 80% of the lower limit of normal without conduction block, at least two sensory nerve action potentials (SNAPs) amplitudes < 80% of the lower limit of normal, and the absence of a decremental response on repetitive nerve stimulation. Exclusion criteria were preexisting neuropathy or myopathy (e.g., amyotrophic lateral sclerosis, Guillain–Barré syndrome, or diabetic peripheral neuropathy), inability to complete neurophysiological examinations (due to edema, fractures, amputations, plaster casts), ICU stay < 7 days [25], elevated serum creatine kinase (CK) levels, or lack of electrophysiological data.

**FIGURE 1:**
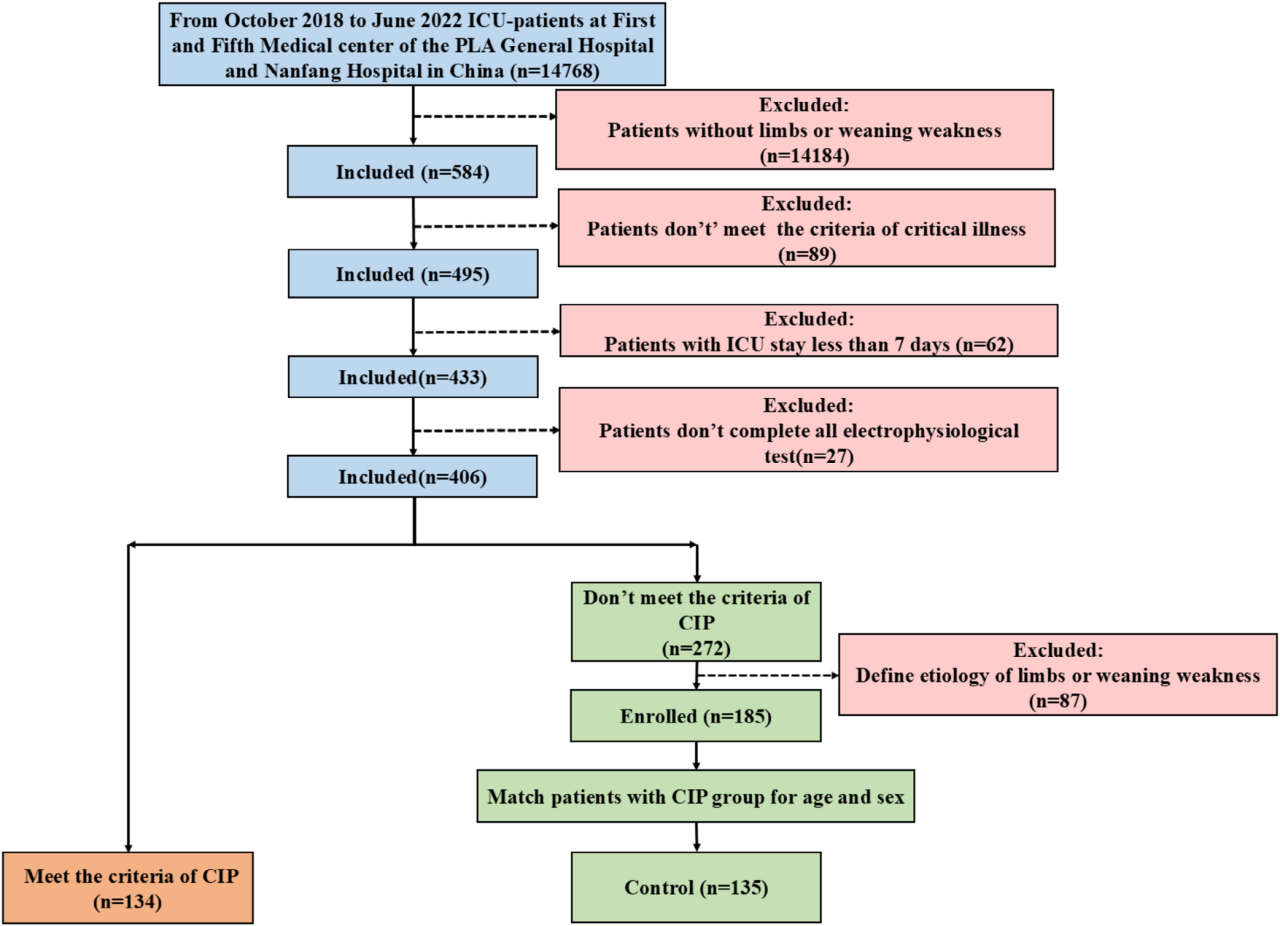
Inclusion and exclusion criteria for patients.

Controls were age‐ and sex‐matched patients who also had organ dysfunction leading to life‐threatening conditions, consistent with the criteria described above, and developed new‐onset limb and/or generalized weakness during ICU admission and weaning that could not be explained by the primary disease (MRC score ≤ 48 or weakness on clinical examination). Exclusion criteria for controls included the inability to complete neurophysiological examinations (e.g., edema, fractures, amputations, and plaster casts), ICU stay < 7 days, or absence of electrophysiological data. Patient selection is summarized in Figure 1.

### 2.2 Electrophysiological Data Acquisition and Preprocessing

All electrophysiological studies were conducted in accordance with the guidelines of the American Association of Neuromuscular and Electrodiagnostic Medicine (AANEM) and followed the standardized protocols described in Electromyography and Neuromuscular Disorders (4th edition, Preston and Shapiro).

Procedures were performed in a dedicated electromyography (EMG) room maintained at approximately 25°C. Prior to testing, distal limb skin temperature was confirmed to be ≥ 32°C to ensure physiological conduction velocities. Patients were positioned in a relaxed supine posture with their limbs comfortably supported to minimize muscle tension and ensure consistency. Recordings were obtained using a Keypoint Workstation (Model 31A06; Alpine Biomed ApS, Denmark) equipped with a high‐resolution analog‐to‐digital converter and a sampling frequency of 10 kHz.

Electrophysiological data were collected using disposable surface electrodes for nerve conduction studies, including one stimulating electrode, one recording electrode, and one ground electrode per tested nerve, as well as disposable concentric needle electrodes (one per muscle) for needle EMG. For motor studies, surface electrodes were placed using a standard belly–tendon montage, with the active electrode (G1) positioned over the muscle belly and the reference electrode (G2) over the distal tendon. For sensory studies, electrodes were placed with G1 closer to the stimulator, maintaining an interelectrode distance of 2.5–4 cm. A disposable ground electrode was positioned between the stimulation and recording sites to minimize electrical noise.

**FIGURE 2:**
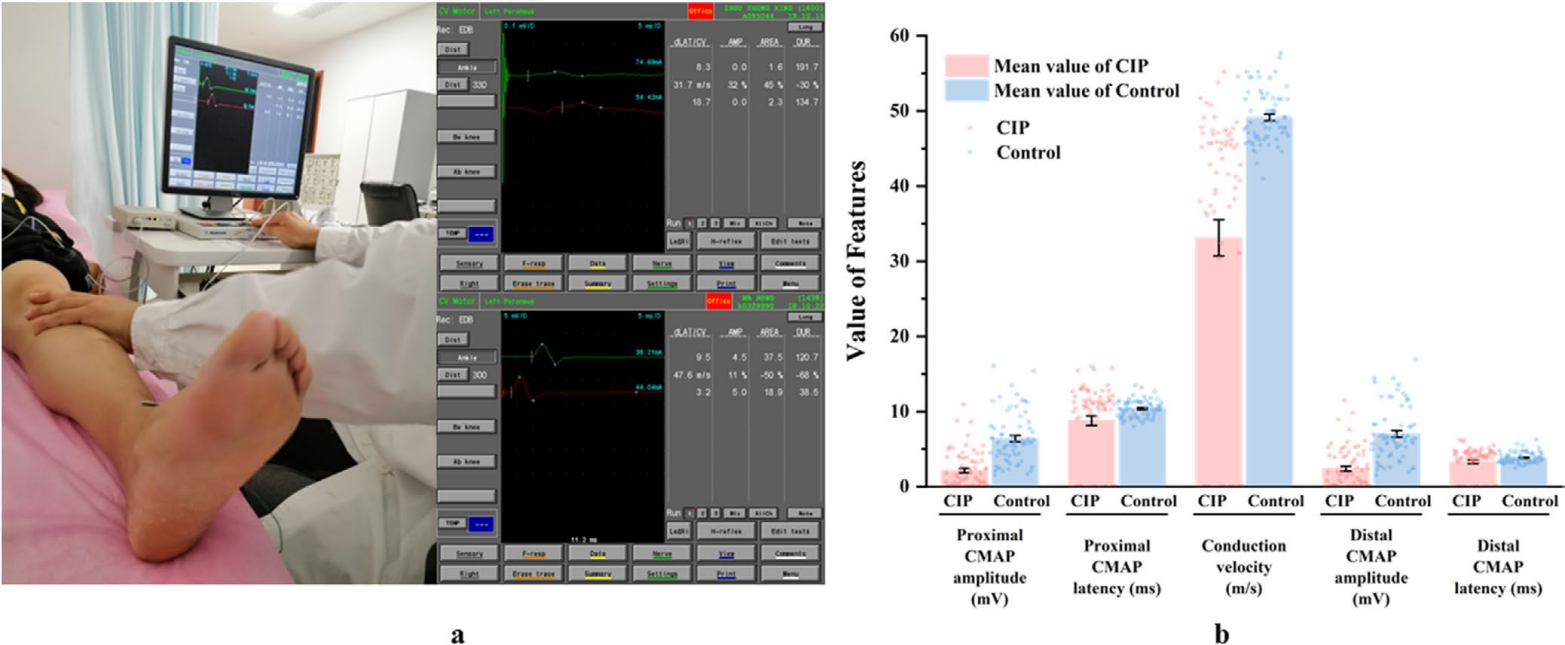
Recording process of nerve conduction and comparison of the proximal CAMP amplitude and latency, distal CAMP amplitude and latency, and conduction velocity of peroneal nerve in patients with CIP and controls. (a) The left side of the figure shows the real‐time recording of nerve conduction electrophysiological data of the left peroneal nerve, the upper right figure shows electrophysiological data of the left peroneal nerve from one of the CIP, and the lower right figure shows electrophysiological data of the left peroneal nerve from one of the control groups. (b) proximal CAMP amplitude and latency, distal CAMP amplitude and latency, and conduction velocity of peroneal nerve in patients with CIP and controls, with pink indicating CIP and light blue indicating the controls.

Electrical stimulation was delivered with a pulse duration of 0.1–0.2 ms and gradually increased to supramaximal levels—typically 20–50 mA for motor studies and 5–30 mA for sensory studies. Representative recordings of peroneal nerve conduction in patients with CIP and controls are shown in Figure 2.

All electrophysiological data were collected and assessed in a blinded manner during the most severe stage of symptoms. Data preprocessing included artifact removal using the Pauta Criterion, followed by normalization with Z‐score standardization.

### 2.3 Electrophysiological Feature Extraction

The electrophysiological features analyzed included proximal and distal CMAP amplitudes (measured from peak to baseline) and latencies, as well as distal conduction velocities of the median, ulnar, tibial, and peroneal nerves. Sensory parameters comprised SNAP amplitudes (anterograde) and conduction velocity, along with sensory latencies of the median, ulnar, and sural nerves. In addition, needle electromyography was performed on the tibialis anterior, quadriceps, first interosseous region, and biceps muscles.

### 2.4 Statistical Analysis

All statistical analyses were performed using SPSS software (version 25.0; SPSS Inc., Chicago, IL, USA), with statistical significance set at *p* < 0.05. Descriptive data were expressed as percentages, medians with interquartile ranges (IQR; 25%–75%), or means ± standard deviations, as appropriate. Categorical variables (e.g., sex, disease type, clinical manifestations, and intubation status) from the clinical and laboratory evaluations were compared using the chi‐square test. The normality of continuous variables was assessed using the Shapiro–Wilk test. Normally distributed data (e.g., red blood cell count [RBC] and hemoglobin [Hb]) were analyzed using independent‐samples *t*‐tests, whereas non‐normally distributed variables (e.g., age, CK, albumin, C‐reactive protein [CRP], glucose, white blood cell [WBC], and interleukin‐6 [IL‐6]) were compared using nonparametric methods, including the Mann–Whitney U test or the Kruskal–Wallis test. Electrophysiological data were evaluated using the Friedman test. For significant results, post hoc pairwise comparisons were conducted using Dunn's test, applying adjustment for multiple comparisons. Pearson's correlation analysis was also performed.

Additionally, samples from enrolled patients and controls were subjected to feature selection using the maximal relevance and minimal redundancy (mRMR) method to evaluate the importance of electrophysiological features in the diagnostic model. Further details regarding mRMR analysis are provided in the Supporting Information.

### 2.5 Machine Learning Models

To establish a rapid diagnostic model, performance was compared with that of a comprehensive model using all available features. The underlying principle was to identify a reduced feature set that achieved diagnostic accuracy compared to that of the full‐feature model. Several widely used machine learning algorithms were employed, including KNN, SVM with radial basis function (SVM‐RBF) and Gaussian kernel function (SVM‐Gauss), RF, and XGB. For reference, these approaches were also compared with conventional nerve conduction studies (NCSs) of the peroneal and sural nerves, which are commonly used in the diagnosis of CIP. All machine learning analyses were performed in MATLAB R2022a. Model parameters were optimized using a grid search strategy, with the parameter set yielding the highest area under the curve (AUC) defined as optimal. Models were trained using five‐fold cross‐validation on the training set. The dataset was randomly divided into training and independent validation sets at a 6:4 ratio, ensuring equal representation of patients with CIP and control subjects.

### 2.6 Performance Criteria

The models were trained using five‐fold cross‐validation, and an independent validation set was applied to assess diagnostic performance. The maximum mean AUC obtained from five‐fold cross‐validation was used to determine the optimal value of the regularization parameter in the training set. To evaluate model performance in both the training and validation sets, mean values of AUC, overall accuracy (ACC), sensitivity (SN), specificity (SP), and precision were calculated. ACC, SN, SP, and precision were defined as follows:

(1) $$\mathrm{ACC}=\frac{\mathrm{TP}+\mathrm{TN}}{\mathrm{TP}+\mathrm{FP}+\mathrm{TN}+\mathrm{FN}}$$

(2) $$\mathrm{SN}=\frac{\mathrm{TP}}{\mathrm{TP}+\mathrm{FN}}$$

(3) $$\mathrm{SP}=\frac{\mathrm{TN}}{\mathrm{TN}+\mathrm{FP}}$$

(4) $$\text{Precision}=\frac{\mathrm{TP}}{\mathrm{TP}+\mathrm{FP}}$$

True positive (TP), false positive (FP), true negative (TN), and false negative (FN) values were obtained by comparing the predicted labels with the actual labels.

**FIGURE 3:**
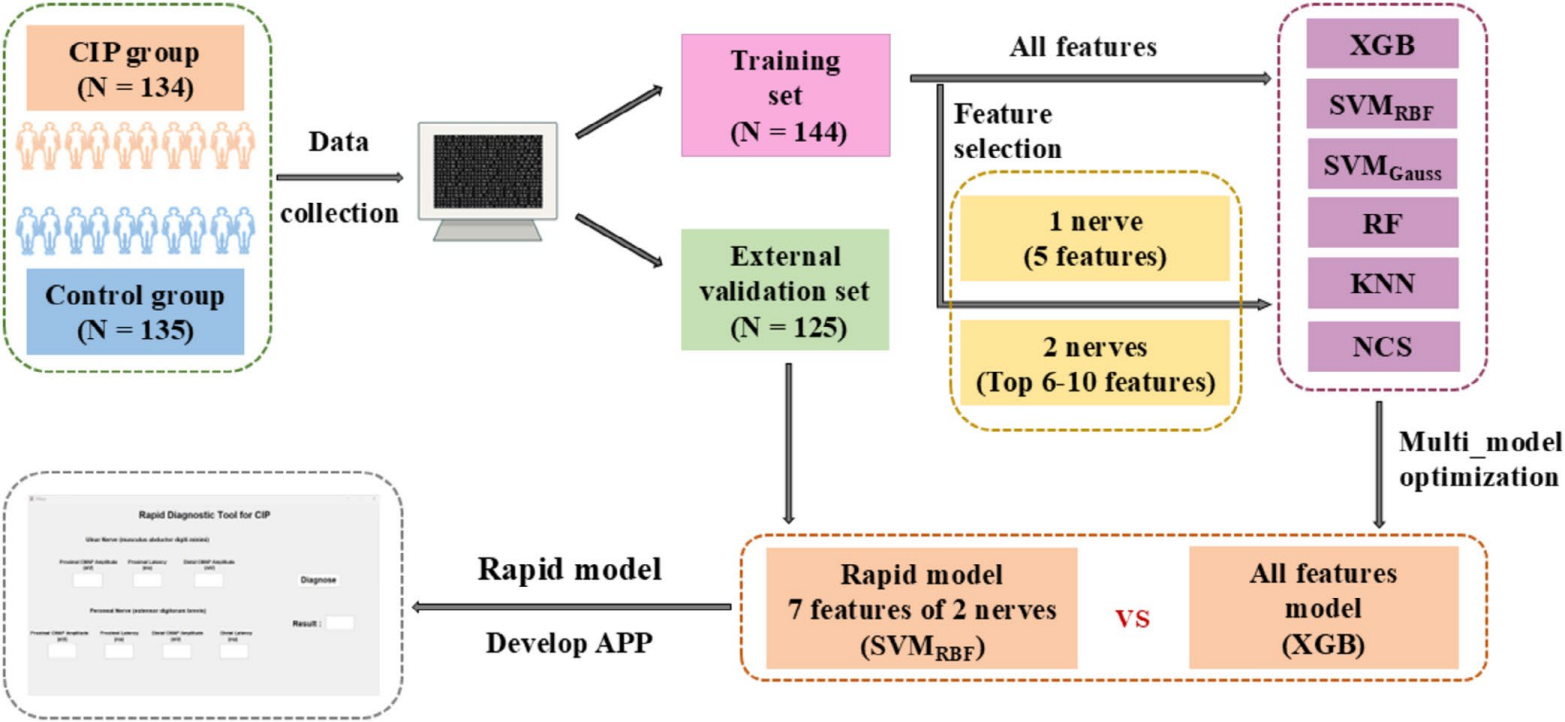
Flowchart of the diagnostic model. First, electrophysiological data, including four sensory nerves, four motor nerves, and three muscles, were obtained from patients with CIP and controls. All the features were subsequently used as inputs to compare the models established by different machine learning methods, and the model with the best performance was selected as the rapid diagnostic model. Meanwhile, the comparison of different feature combination models is used to optimize the diagnostic model. Finally, the feature combination with the best performance and the most similar to the all‐feature model was selected to establish the final rapid diagnostic model, and the app was developed.

**FIGURE 4:**
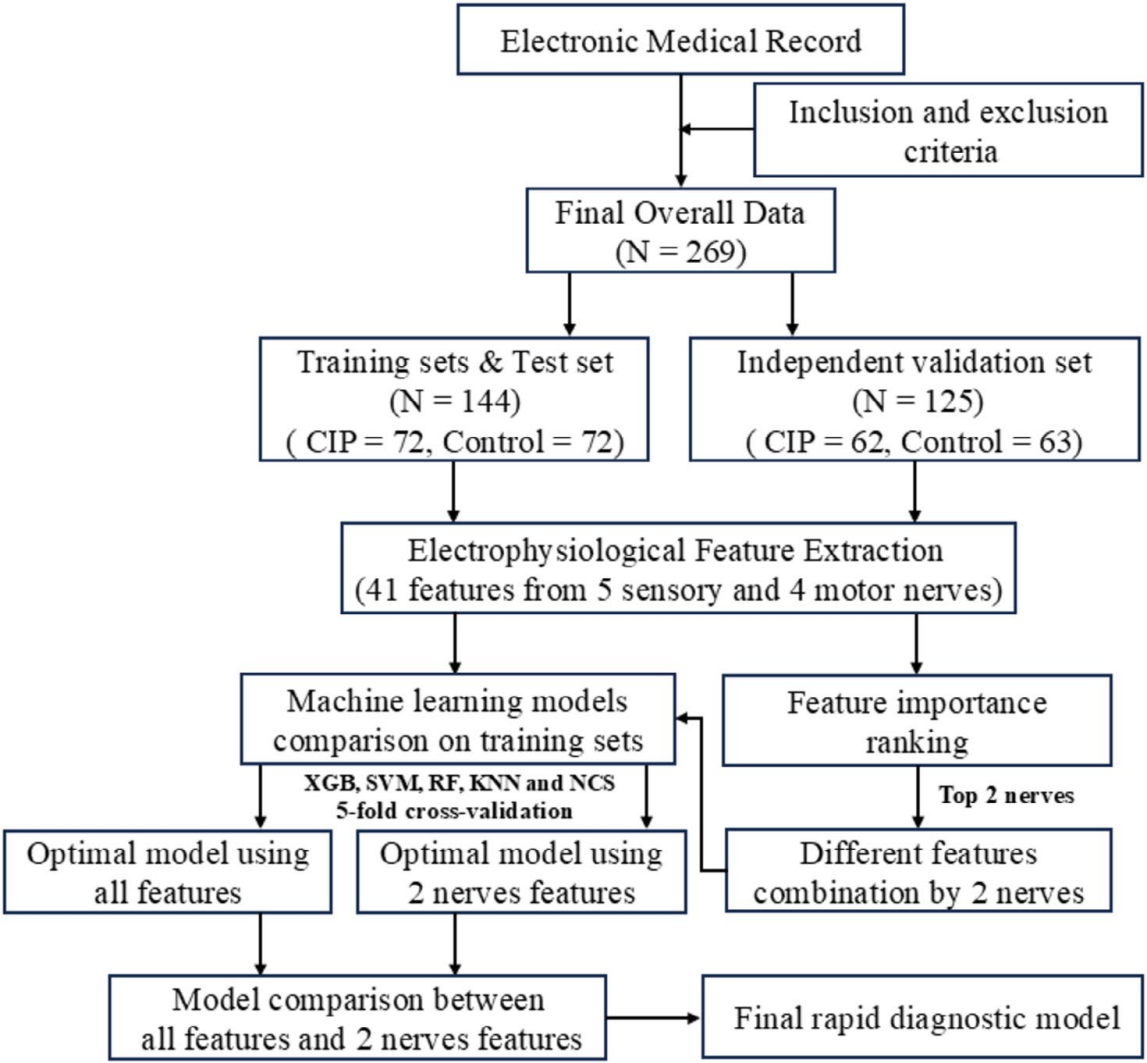
Computational steps of the proposed algorithm.

A flowchart of the rapid diagnostic model is presented in Figure 3, and the computational steps of the proposed algorithm are illustrated in Figure 4. All applications were developed and implemented in MATLAB 2016a.

## 3 Results

### 3.1 Demographic and Clinical Data

Between October 2018 and June 2022, a total of 14,768 patients admitted to the ICUs of the First Medical Center, Fifth Medical Center of the PLA General Hospital, and Nanfang Hospital were screened (Figure 1). Based on the inclusion and exclusion criteria, 134 patients with CIP and 135 controls were enrolled. Of these, 72 patients with CIP and 72 controls at the First Medical Center were randomly assigned to the training set. The independent multicenter validation set comprised 29 patients with CIP and 29 controls from the First Medical Center, 15 patients with CIP and 15 controls from the Fifth Medical Center, and 18 patients with CIP and 19 controls from Nanfang Hospital. In total, 144 subjects were included in the training set and 125 in the validation set.

The predominant underlying etiology among enrolled patients was infection (44.03%), and most cases presented limb weakness (84.33%). There were no statistically significant differences between the CIP and control groups with respect to age, sex, disease type, clinical manifestations, or intubation status. Laboratory indices showed that patients with CIP had significantly lower albumin levels (*p* < 0.05) and higher IL‐6 levels (*p* < 0.05) compared with controls. RBC counts and Hb levels were lower in the patient group, but the differences were not statistically significant. WBC counts and CRP, CK, and glucose levels were also higher in the patient group, though not significantly different from controls. Detailed demographic, clinical, and laboratory characteristics are presented in Table 1. The MRC score was reduced in the distal limbs (median proximal upper limb: 3 [IQR 1–4]; median distal upper limb: 2.5 [IQR 1–3.5]; median proximal lower limb: 3 [IQR 1–3.5]; and median distal lower limb: 2 [IQR 1–3]). A summary of these results is shown in Table 2. Results of the normality tests and quantile–quantile plots for all variables are provided in Table S1 and Figure S1 of the Supporting Information.

**TABLE 1:** Characteristics of the patients and controls in this study.

Clinical features	Patients with CIP (*n* = 134)	Controls (*n* = 135)	*p*
Age (years)	57.00 [38 69]	56.50 [39 67]	0.613
Sex			0.154
Male	89 (66.42%)	76 (56.30%)	
Female	45 (33.58%)	59 (43.70%)	
Disease			0.158
Pulmonary infection	23 (17.16%)	12 (8.89%)	
Organ failure	20 (14.93%)	17 (12.59%)	
Intracranial infection	36 (26.87%)	46 (34.07%)	
Cerebrovascular diseases	25 (18.66%)	24 (17.78%)	
Autoimmune encephalitis	15 (11.19%)	13 (9.63%)	
Demyelination in CNS	13 (9.70%)	12 (8.89%)	
Others	2 (1.49%)	11 (8.15%)	
Clinical manifestations			0.194
Weakness of limbs	113 (84.33%)	114 (84.44%)	
Weakness during weaning	7 (5.22%)	2 (1.48%)	
Both	14 (10.45%)	19 (14.07%)	
Intubated	93 (69.40%)	89 (65.93%)	0.582
Creatine Kinase (U/L)	50.90 (24.00, 93.70)	59.70 (35.00, 82.50)	0.2402
Albumin (g/L)	34.50 (31.90, 38.10)	40.30 (34.80, 44.40)	< 0.05
RBC (10^12^/L)	3.52 ± 0.75	3.61 ± 0.62	0.337
Hb (g/L)	107.06 ± 21.54	111.61 ± 28.31	0.199
CRP (mg/L)	1.78 (0.92, 2.95)	1.37 (0.45, 3.63)	0.357
Glucose (mmol/L)	7.35 (5.88, 10.08)	7.32 (5.47, 9.03)	0.051
WBC (10^9^/L)	7.99 (6.11, 10.71)	7.23 (5.57, 9.40)	0.426
IL‐6 (pg/mL)	18.69 (8.82, 39.42)	13.47 (7.17, 22.24)	< 0.05

*Note:* Variables following a normal distribution (RBC, Hb) are presented as mean ± standard deviation, whereas non‐normally distributed continuous variables (Age, Creatinine Kinase, Albumin, CRP, Glucose, WBC, IL‐6) are summarized as medians (IQRs). Categorical variables (Sex, Disease, Clinical manifestations, and intubation) are summarized as numbers and percentages.

**TABLE 2:** The MRC of the 101 patients enrolled in the study.

Clinical features	CIP‐patient
MRC score (*N* = 101)	33 [12 42]
Upper distal limbs (*N* = 202)	2.5 [1 3.5]
Lower distal limbs (*N* = 202)	2 [1 3]
Upper proximal limbs (*N* = 606)	3 [1 4]
Lower proximal limbs (*N* = 606)	3 [1 3.5]

### 3.2 Electrophysiological Data

A total of 41 electrophysiological features were collected. These included: (1) distal and proximal latencies, distal and proximal CMAP amplitudes, and motor conduction velocity (MCV) values of the median (motor), ulnar (motor), tibial, and peroneal nerves; (2) latencies, SNAP amplitudes, and sensory conduction velocity (SCV) values of the median (sensory), ulnar (sensory), and sural nerves; and (3) Needle EMG parameters, including insertional activity, positive sharp waves, and fibrillation potentials of the first dorsal interosseous, biceps, tibialis anterior, and quadriceps muscles.

#### 3.2.1 Electrophysiological Features

**FIGURE 5:**
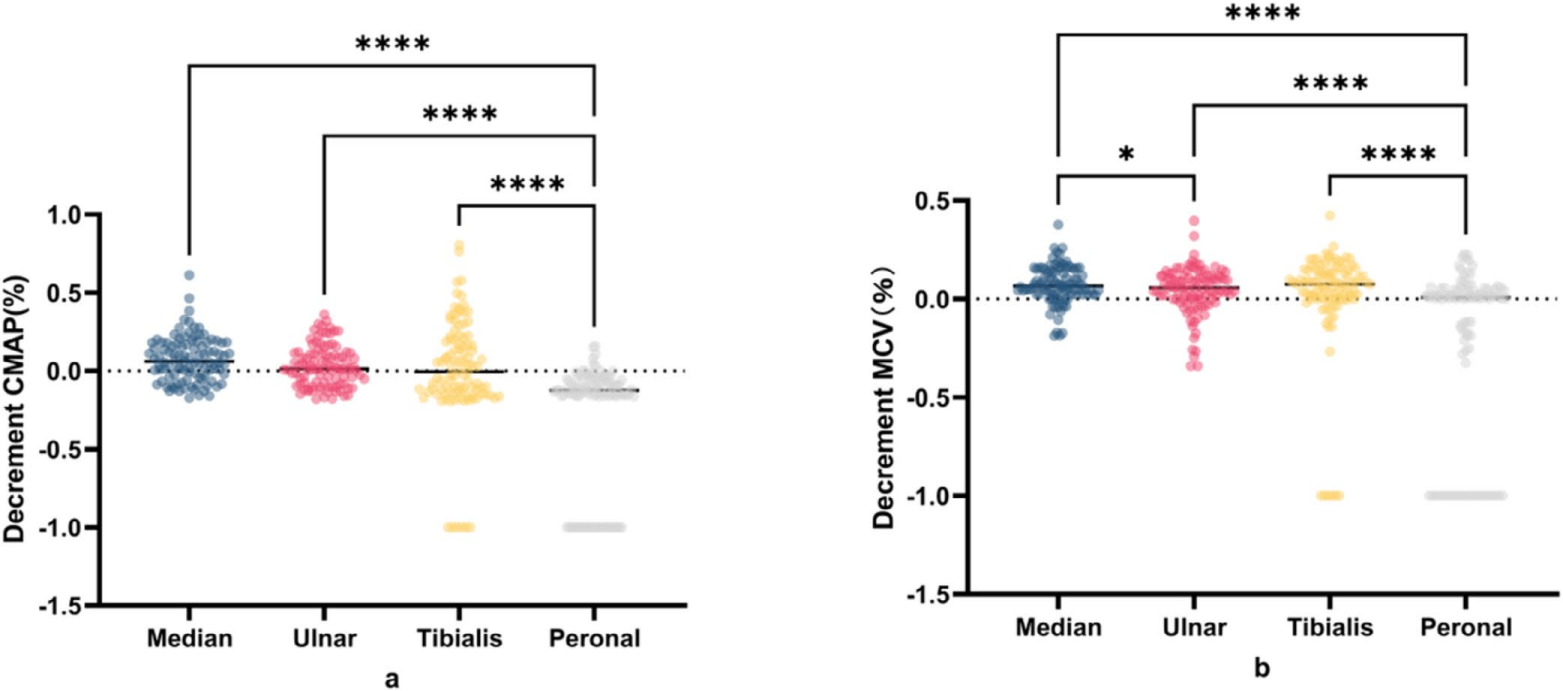
CMAP and MCV of patients with CIP. (a) CMAP decrement in four nerves (median, ulnar, tibialis, and peroneal) of patients with CIP (median vs. peroneal, *F* = 180.5, *p* < 0.0001; ulnar vs. peroneal, *F* = 153, *p* < 0.0001; and tibialis vs. peroneal, *F* = 148.5, *p* < 0.0001). (b) Decreases in the MCVs of four nerves (median, ulnar, tibialis, and peroneal) in patients with CIP (median vs. peroneal, *F* = 146, *p* < 0.0001; median vs. ulnar, *F* = 52, *p* = 0.0276; ulnar vs. peroneal, F = 94, *p* < 0.0001; and tibialis vs. peroneal, F = 112, *p* < 0.0001). CIP, critical illness polyneuropathy; CMAP, compound muscle action potential; MCV, motor conduction velocity; SCV, sensory conduction velocity; SNAP, sensory nerve action potential.

In motor nerve conduction tests, the peroneal nerve exhibited the highest abnormality rate (92.1%). The reduction in distal CMAP amplitude of the peroneal nerve was significantly greater than that observed in the other three motor nerves (*F* = 119.9, *p* < 0.0001; median vs. peroneal, *F* = 180.5, *p* < 0.0001; ulnar vs. peroneal, *F* = 153, *p* < 0.0001; and tibialis vs. peroneal, *F* = 148.5, *p* < 0.0001). Similarly, the MCV of the peroneal nerve significantly differed from those of the other motor nerves (*F* = 70.32, *p* < 0.0001; median vs. peroneal, *F* = 146, *p* < 0.0001; median vs. ulnar, *F* = 52, *p* = 0.0276; ulnar vs. peroneal, *F* = 94, *p* < 0.0001; and tibialis vs. peroneal, *F* = 112, *p* < 0.0001). These results are shown in Figure 5, and detailed values are provided in Tables 3 and 4.

**TABLE 3:** Electrophysiological data of different nerves.

Electrophysiological features	Patients with CIP (*n* = 134)	Controls (*n* = 135)
Motor nerve‐Distal CMAP		
Median	6.84 ± 3.56	12.93 ± 4.30
Ulnar	5.80 ± 3.24	11.29 ± 2.82
Tibialis	6.72 ± 5.78	14.90 ± 6.25
Peroneal	3.02 ± 2.66	6.87 ± 3.48
Motor nerve‐Proximal CMAP		
Median	6.35 ± 3.44	12.13 ± 4.05
Ulnar	5.29 ± 3.11	10.49 ± 2.80
Tibialis	5.64 ± 5.10	12.20 ± 5.08
Peroneal	2.68 ± 2.40	6.26 ± 3.24
Motor nerve‐MCV		
Median	53.54 ± 4.74	59.11 ± 4.69
Ulnar	52.09 ± 6.06	58.33 ± 5.15
Tibialis	43.07 ± 4.05	46.75 ± 3.52
Peroneal	45.41 ± 5.11	49.16 ± 3.62
Sensory nerve‐SNAP		
Median	2.27 ± 1.27	5.75 ± 2.30
Ulnar	1.88 ± 0.63	5.34 ± 2.14
Sural	2.09 ± 0.97	9.08 ± 3.35
Sensory nerve‐SCV		
Median	53.83 ± 4.59	59.39 ± 4.33
Ulnar	53.60 ± 4.69	56.53 ± 4.30
Sural	54.67 ± 5.12	57.71 ± 5.72

Abbreviations: CMAP, compound muscle action potential; MCV, motor conduction velocity; SCV, sensory conduction velocity; SNAP, sensory nerve action potential.

**TABLE 4:** The abnormality rate of different nerves.

Object	Median (*n*, %)	Ulnar (*n*, %)	Tibial (*n*, %)	Peroneal (*n*, %)	Sural (*n*, %)
CMAP					
Abnormal	30 (29.70)	41 (40.59)	51 (50.50)	93 (92.1)	N/A
Normal	71 (70.30)	60 (59.41)	50 (49.50)	8 (7.92)	N/A
MCV					
Abnormal	20 (19.80)	31 (30.69)	28 (27.72)	38 (37.62)	N/A
Normal	81 (80.19)	70 (69.31)	73 (72.28)	63 (62.38)	N/A
SNAP					
Abnormal	32 (31.68)	80 (79.21)	N/A	N/A	78 (77.23)
Normal	69 (68.32)	21 (20.79)	N/A	N/A	23 (22.77)
SCV					
Abnormal	24 (23.76)	31 (30.69)	N/A	N/A	32 (31.68)
Normal	77 (76.24)	70 (69.31)	N/A	N/A	69 (68.32)

Abbreviations: CMAP, compound muscle action potential; MCV, motor conduction velocity; N/A, not applicable; SCV, sensory conduction velocity; SNAP, sensory nerve action potential.

#### 3.2.2 Feature Selection

The mRMR algorithm was used to evaluate the importance of electrophysiological features in CIP diagnosis. This approach, based on mutual information (MI), assesses both the correlation of features with the target labels and the redundancy among features already selected. In this study, the importance of all the features was evaluated, and their rankings obtained through mRMR applied to the training set are presented in Table S2.

#### 3.2.3 Performance Comparison of Machine Learning Models

**FIGURE 6:**
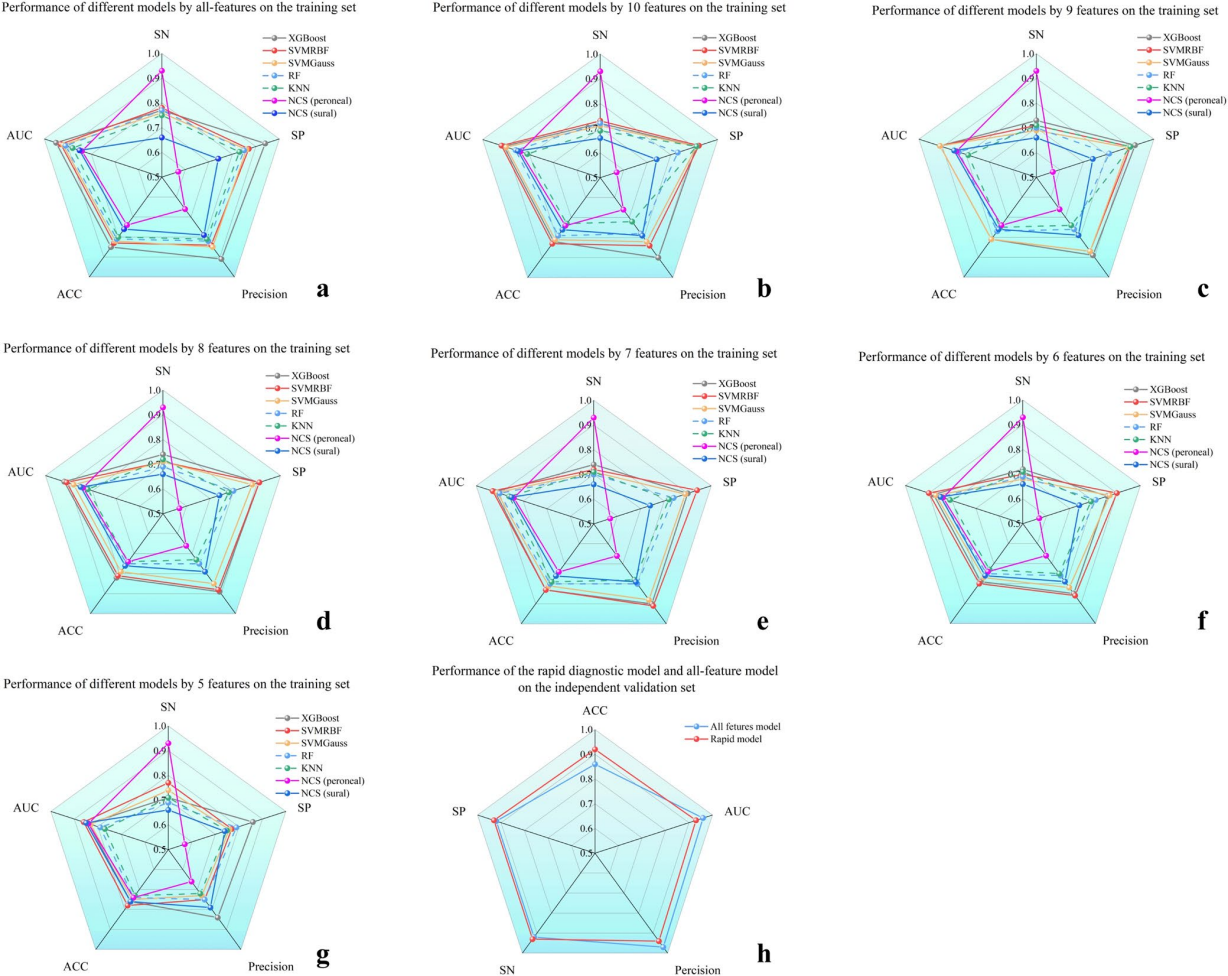
Performance of different machine learning models and feature combinations on the training set or independent validation set via 5‐fold cross‐validation. (a) Performance of 5‐fold cross‐validation experiments of different machine learning models with all features on the training set. Mean AUC (XGB) = 0.95, mean AUC (SVM‐RBF) = 0.93, mean AUC (SVM‐Gauss) = 0.92, mean AUC (RF) = 0.91, mean AUC (KNN) = 0.88, mean AUC (NCS (peroneal)) = 0.84, mean AUC (NCS (sural)) = 0.85, (b) Performance of 5‐fold cross‐validation experiments of different models with 10 features on the training set. Mean AUC (XGB) = 0.91, mean AUC (SVM‐RBF) = 0.92, mean AUC (SVM‐Gauss) = 0.90, mean AUC (RF) = 0.86, mean AUC (KNN) = 0.81, mean AUC (NCS (peroneal)) = 0.84, mean AUC (NCS (sural)) = 0.85, (c) Performance of 5‐fold cross‐validation experiments of different models with nine features on the training set. Mean AUC (XGB) = 0.91, mean AUC (SVM‐RBF) = 0.91, mean AUC (SVM‐Gauss) = 0.91, mean AUC (RF) = 0.83, mean AUC (KNN) = 0.79, mean AUC (NCS (peroneal)) = 0.84, mean AUC (NCS (sural)) = 0.85, (d) Performance of 5‐fold cross‐validation experiments of different models with eight features on the training set. Mean AUC (XGB) = 0.92, mean AUC (SVM‐RBF) = 0.91, mean AUC (SVM‐Gauss) = 0.88, mean AUC (RF) = 0.84, mean AUC (KNN) = 0.82, mean AUC (NCS [peroneal]) = 0.84, mean AUC (NCS [sural]) = 0.85, (e) Performance of 5‐fold cross‐validation experiments of different models with seven features on the training set. Mean AUC (XGB) = 0.92, mean AUC (SVM‐RBF) = 0.93, mean AUC (SVM‐Gauss) = 0.91, mean AUC (RF) = 0.90, mean AUC (KNN) = 0.86, mean AUC (NCS [peroneal]) = 0.84, mean AUC (NCS [sural]) = 0.85, (f) Performance of 5‐fold cross‐validation experiments of different models with six features on the training set. Mean AUC (XGB) = 0.88, mean AUC (SVM‐RBF) = 0.90, mean AUC (SVM‐Gauss) = 0.87, mean AUC (RF) = 0.85, mean AUC (KNN) = 0.81, mean AUC (NCS [peroneal]) = 0.84, mean AUC (NCS [sural]) = 0.85, (g) Performance of 5‐fold cross‐validation experiments of different models with five features on the training set. Mean AUC (XGB) = 0.84, mean AUC (SVM‐RBF) = 0.86, mean AUC (SVM‐Gauss) = 0.83, mean AUC (RF) = 0.79, mean AUC (KNN) = 0.77, mean AUC (NCS [peroneal]) = 0.84, mean AUC (NCS [sural]) = 0.85, (h) Performance of the rapid diagnostic model and all‐feature model on the independent validation set, mean AUC (All‐features) = 0.90, mean AUC (Rapid diagnostic model) = 0.88.

The XGB, SVM, RF, and KNN models were compared with peroneal and sural nerves NCSs in the training set using five‐fold cross‐validation. For the XGB, SVM, and RF models, all electrophysiological features were included. The comparative test results for the five models are shown in Figure 6a. The results demonstrated that the XGB model outperformed the SVM and RF models across all evaluation metrics. Specifically, the XGB model achieved SN of 0.77, SP of 0.94, precision of 0.91, ACC of 0.85, and AUC of 0.95. These values consistently exceeded those obtained from NCSs of the peroneal and sural nerves, with AUC improvements of 9% and 13%, respectively. Among the tested machine learning approaches, the XGB model demonstrated superior performance and was identified as the optimal choice.

### 3.3 Diagnostic Models With Different Feature Combinations

Based on the feature ranking obtained from the mRMR algorithm, the peroneal and ulnar nerves were consistently among the top 20 contributors. Therefore, electrophysiological data from these nerves were selected to construct a rapid diagnostic model for optimal comparison. When a single nerve was required, features from the peroneal nerve were chosen, as its indicator rankings were higher than those of the ulnar nerve. Recursive feature elimination was applied by gradually removing the lowest‐ranked features of the peroneal and ulnar nerves (from the top 10 to the top 6) to determine the best feature combination. Models were evaluated on the training set by five‐fold cross‐validation, with the model yielding the highest AUC identified as optimal. The results of these models are shown in Figure 6b–f, with additional details provided in Tables S3–S9.

Among the seven different feature combinations, the XGB model using all features achieved the best overall performance, with an ACC of 0.85 and an AUC of 0.95. The SVM‐RBF model using seven features (proximal latency, distal latency, and CMAP amplitude of the peroneal nerve, along with proximal latency, CMAP amplitude, and distal CMAP amplitude of the ulnar nerve) achieved performance most similar to the all‐feature model. This model yielded an ACC of 0.83, an AUC of 0.83, and the highest SP of 0.94 and precision of 0.91. In contrast, the model using five features from the peroneal nerve alone achieved the highest SN (0.77) but had the lowest values across the other evaluation metrics.

### 3.4 Performance of the Independent Validation Set

The performance of the independent validation set, compared with the model using all features, is shown in Figure 6h and detailed in Table S10. The all‐feature model achieved an AUC of 0.90, while the rapid diagnostic model using seven features achieved an AUC of 0.88.

**FIGURE 7:**
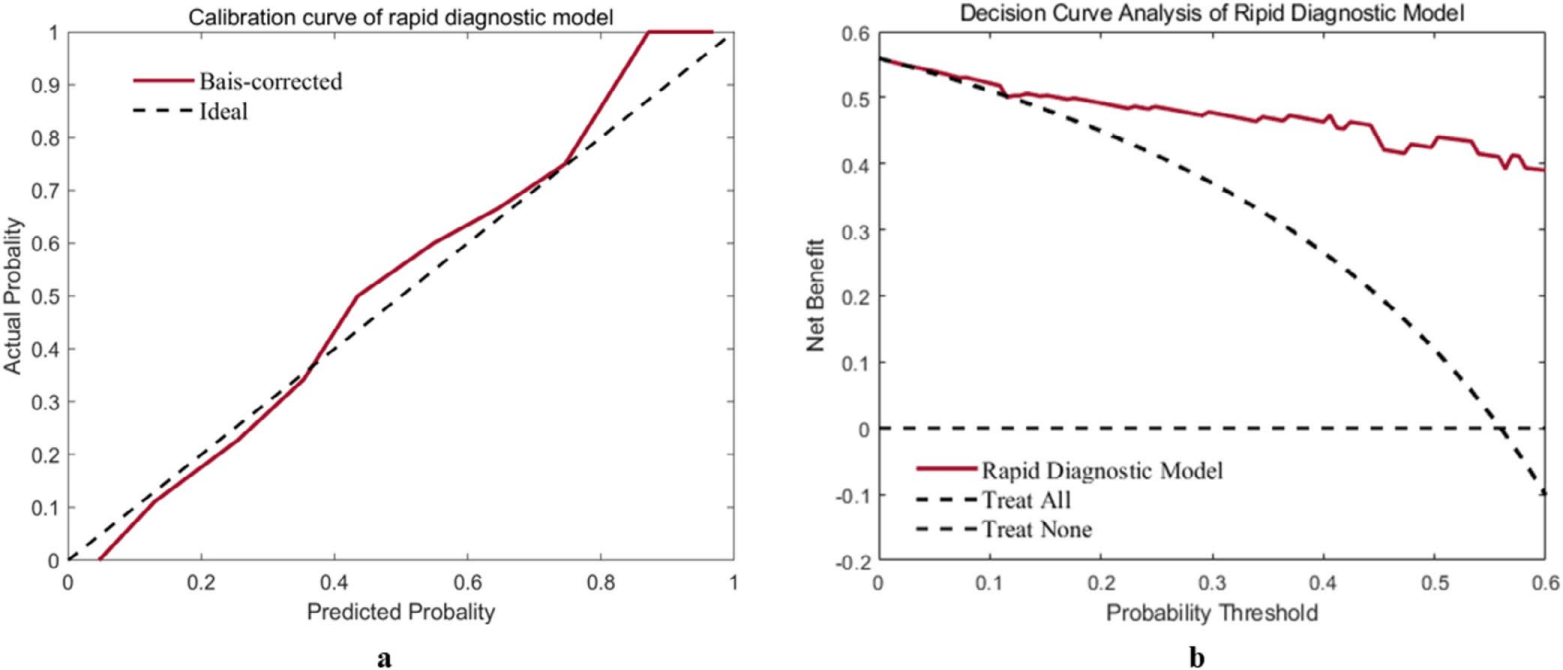
The calibration curve and clinical decision curve analysis of rapid diagnostic model. (a) Calibration curve of rapid diagnostic model. Absolute error = 0.042, *n* = 125. (b) Decision curve analysis of rapid diagnostic model. For the rapid diagnostic model, the net benefit curve is shown. Gray dotted line = net benefit when all patients with CIP are considered as not having the outcome; Black dotted line = net benefit when all patients with CIP are considered as having the outcome.

Calibration curve analysis and clinical decision curve (DCA) were performed for the rapid diagnostic model to objectively quantify its net benefit across varying decision thresholds and to evaluate its potential for risk stratification in real‐world clinical settings. The results are presented in Figure 7. The final model was developed in MATLAB 2022a, and its software interface is shown in Figure S2.

## 4 Discussion

The prognosis of patients with CIP is generally poor, underscoring the need for a rapid diagnostic model. To address this, we constructed a machine learning–based model using electrophysiological data. In this study, patient selection was guided by established inclusion criteria for ICUAW, with individuals having non‐critical illnesses excluded. As reported in previous literature, CIP typically develops after approximately 7 days in critically ill patients [8, 25, 26]; therefore, patients admitted to the ICU for fewer than 7 days were excluded.

The prevalence of CIP in this cohort was 23.2%, which is lower than the approximately 30% or higher prevalence reported in prior studies, where variability often depended on the underlying disease condition [24]. This relatively low prevalence may be explained by the fact that many of our patients had neurological diseases (e.g., cerebrovascular diseases, etc.) accompanied by only a mild systemic inflammatory response. Infectious diseases were the most common primary condition among enrolled patients, supporting previous findings that infections increase susceptibility to CIP. This is consistent with earlier reports demonstrating that CIP frequently occurs in conjunction with infectious encephalopathy [27, 28, 29]. Furthermore, our results confirmed that CIP primarily manifests as weakness of the extremities, with distal lower limb involvement being most prominent. The MRC scores also indicated greater weakness in the distal or lower extremities compared with the proximal or upper extremities [30]. Therefore, when critically ill patients present with bilateral lower‐extremity weakness and dyspnea, the possibility of CIP should be considered. Previous studies have suggested that ICUAW often manifests as proximal weakness [26]; however, our findings indicate that if proximal weakness is observed in clinical practice, CIP should not be the initial consideration. Instead, CIM or CIPNM may be more likely. Laboratory test results in this study revealed only slight elevations in blood glucose, IL‐6, and CRP levels, with no significant abnormalities. Prior reports have identified hyperglycemia, parenteral nutrition, disease severity, and hypoproteinemia as risk factors for CIP [10, 31, 32, 33, 34, 35, 36], while elevated CK levels are frequently observed in patients with CIPNM and CIM. Our results indicated that the severity of laboratory abnormalities may not correlate with CIP severity but rather with its occurrence (Figure S3).

The electrophysiological findings of this study demonstrated that the peroneal nerve exhibited the highest abnormality rate, with the distal CMAP amplitude contributing most strongly to CIP diagnosis based on feature ranking. This finding is consistent with the clinical presentation of symmetrical distal limb weakness in patients with CIP [12, 13, 14]. The high abnormality rate of the peroneal nerve may be explained by its long course and increased vulnerability to ischemia [13]. Previous research has shown that a unilateral peroneal nerve–based model using receiver operating characteristic (ROC) curves can serve as an initial screening tool for CIPNM, achieving 94% sensitivity and 74% specificity [14]. These results have been further supported by subsequent studies [13]. While peroneal nerve testing may therefore be valuable as an initial screening method, reliance on a single nerve is limited by susceptibility to confounding factors, such as postoperative unilateral peroneal nerve palsy, which can reduce diagnostic specificity. Accordingly, the development of rapid and accurate diagnostic models incorporating multiple electrophysiological features is essential.

In this study, we compared the diagnostic performance of peroneal and sural nerve NCS‐based models with several machine learning approaches, including XGB, SVM‐RBF, SVM‐Gauss, KNN, and RF. The unilateral peroneal nerve model demonstrated higher SN (0.93) but lower ACC (0.74), AUC (0.77) compared with the machine learning model (ACC = 0.81–0.83, AUC = 0.84–0.93). Among the latter, the XGB model achieved the best diagnostic performance (ACC = 0.85, AUC = 0.95). Given that comprehensive electrophysiological testing is often limited by patient cooperation, operator workload, and time requirements, simplifying the set of features needed for accurate diagnosis is of critical importance. Needle EMG is further constrained by operator expertise [37], while venous or arterial catheters may interfere with measurement of the median and radial nerves [14]. Additionally, sensory nerve conduction studies are frequently affected by edema and other factors that reduce accuracy. For these reasons, motor nerve parameters were selected as the basis for development in this study. By combining the top 20 features, the most frequently represented parameters were derived from the peroneal nerve and ulnar motor nerve (3 of 20). Accordingly, 10 features from these two nerves were evaluated, and models were constructed by sequentially removing the lowest‐ranked features. A seven‐feature model achieved performance comparable to that of the all‐feature model. Specifically, the SVM‐RBF model based on seven features from the peroneal and ulnar nerves demonstrated superior accuracy (ACC = 0.83, AUC = 0.93) compared with the model using only peroneal nerve features (ACC = 0.78, AUC = 0.86). The calibration curve of the rapid diagnostic model indicated good agreement with the observed outcomes, while DCA confirmed strong clinical adaptability. Therefore, a model based on seven features from two nerves was established, offering greater diagnostic utility than alternative models. Importantly, this approach reduced diagnostic time to approximately 10–20 min, compared with approximately 90 min required for conventional testing.

## 5 Limitations

This diagnostic model offers several advantages: it is convenient, accurate, minimally invasive for patients, and less technically demanding for examiners, factors that are particularly important in the diagnosis of CIP. However, certain limitations should be noted. First, further studies with larger sample sizes are required to validate the model, ideally through larger, multicenter studies involving multidisciplinary teams. Second, while the simplified peroneal nerve–based approach has been useful, it demonstrates relatively low diagnostic accuracy in pediatric ICUAW. Whether our model can be reliably extended to pediatric populations remains to be determined.

## 6 Conclusions

In this study, we developed a rapid diagnostic model for CIP in ICU patients using seven electrophysiological indices from the peroneal and ulnar nerves with an SVM‐RBF approach. This model demonstrated superior diagnostic accuracy compared with both all‐feature and single‐nerve models. Importantly, it reduced diagnostic time to 10–20 min, representing a substantial improvement over conventional testing (60–90 min). These results support the clinical utility of this model and highlight its potential to facilitate timely diagnosis and treatment of patients with CIP.

## Author Contributions

Y.L., Z.Z., S.Y. and F.Y. had full access to all study data and take responsibility for the integrity of the data analysis. Y.L., Z.Z., S.Y. and F.Y. conceptualized and designed the study. All authors contributed to the analysis and/or interpretation of the data. Y.L. Z.Z. and Z.J. drafted the original manuscript. S.Y. and F.Y. critically revised the manuscript for important intellectual content.

## Ethics Statement

This study was approved by the Ethics Committee of Chinese PLA General Hospital (S2022‐706‐01). All patients were involved in the study based on the voluntary principle.

## Consent

The authors have nothing to report.

## Conflicts of Interest

The authors declare no conflicts of interest.

## Supporting information


**Data S1:** cns70631‐sup‐0001‐Supinfo.docx.

## Data Availability

A de‐identified data set and the study protocol may be made available to researchers with a methodologically sound proposal to achieve the aims described in the approved proposal. Data will be available upon request following article publication. Requests for data should be directed at yangfei@301hospital.com.cn to gain access.

## References

[1] R. D. Stevens , S. A. Marshall , D. R. Cornblath , et al., “A Framework for Diagnosing and Classifying Intensive Care Unit‐Acquired Weakness,” Critical Care Medicine 37, no. SUPPL. 10 (2009): S299–S308, 10.1097/CCM.0b013e3181b6ef67. 20046114

[2] E. Fan , F. Cheek , L. Chlan , et al., “An Official American Thoracic Society Clinical Practice Guideline: The Diagnosis of Intensive Care Unit‐Acquired Weakness in Adults,” American Journal of Respiratory and Critical Care Medicine 190, no. 12 (2014): 1437–1446, 10.1164/rccm.201411-2011ST. 25496103

[3] C. F. Bolton , J. J. Gilbert , A. F. Hahn , and W. J. Sibbald , “Polyneuropathy in Critically Ill Patients,” Journal of Neurology, Neurosurgery & Psychiatry 47, no. 11 (1984): 1223–1231, 10.1136/jnnp.47.11.1223. 6094735 PMC1028091

[4] J. Bednarik , Z. Lukas , P. Vondracek , and Critical illness polyneuromyopathy , “The Electrophysiological Components of a Complex Entity,” Intensive Care Medicine 29, no. 9 (2003): 1505–1514, 10.1007/s00134-003-1858-0. 12879242

[5] H. Lad , T. M. Saumur , M. S. Herridge , et al., “Intensive Care Unit‐Acquired Weakness: Not Just Another Muscle Atrophying Condition,” International Journal of Molecular Sciences 21, no. 21 (2020): 7840, 10.3390/ijms21217840. 33105809 PMC7660068

[6] C. L. Hough , B. K. Lieu , and E. S. Caldwell , “Manual Muscle Strength Testing of Critically Ill Patients: Feasibility and Interobserver Agreement,” Critical Care 15, no. 1 (2011): R43, 10.1186/cc10005. 21276225 PMC3221972

[7] J. P. Kress and J. B. Hall , “ICU‐Acquired Weakness and Recovery From Critical Illness,” New England Journal of Medicine 370, no. 17 (2014): 1626–1635, 10.1056/NEJMra1209390. 24758618

[8] B. De Jonghe , T. Sharshar , J. P. Lefaucheur , et al., “Paresis Acquired in the Intensive Care Unit: A Prospective Multicenter Study,” Journal of the American Medical Association 288, no. 22 (2002): 2859–2867, 10.1001/jama.288.22.2859. 12472328

[9] J. Khan , T. B. Harrison , M. M. Rich , and M. Moss , “Early Development of Critical Illness Myopathy and Neuropathy in Patients With Severe Sepsis,” Neurology 67, no. 8 (2006): 1421–1425, 10.1212/01.wnl.0000239826.63523.8e. 17060568

[10] G. Hermans , A. Wilmer , W. Meersseman , et al., “Impact of Intensive Insulin Therapy on Neuromuscular Complications and Ventilator Dependency in the Medical Intensive Care Unit,” American Journal of Respiratory and Critical Care Medicine 175, no. 5 (2007): 480–489, 10.1164/rccm.200605-665OC. 17138955

[11] G. Hermans , H. Van Mechelen , F. Bruyninckx , et al., “Predictive Value for Weakness and 1‐Year Mortality of Screening Electrophysiology Tests in the ICU,” Intensive Care Medicine 41, no. 12 (2015): 2138–2148, 10.1007/s00134-015-3979-7. 26266842

[12] N. Latronico , G. Bertolini , B. Guarneri , et al., “Simplified Electrophysiological Evaluation of Peripheral Nerves in Critically Ill Patients: The Italian Multi‐Centre CRIMYNE Study,” Critical Care 11, no. 1 (2007): 1111, 10.1186/cc5671. PMC2151880 17254336

[13] N. Latronico , G. Nattino , B. Guarneri , N. Fagoni , A. Amantini , and G. Bertolini , “Validation of the Peroneal Nerve Test to Diagnose Critical Illness Polyneuropathy and Myopathy in the Intensive Care Unit: The Multicentre Italian CRIMYNE‐2 Diagnostic Accuracy Study,” F1000Research 3, no. 127 (2014): 33933.3, 10.12688/f1000research.3933.3. PMC4184363 25309729

[14] M. Moss , M. Yang , M. Macht , et al., “Screening for Critical Illness Polyneuromyopathy With Single Nerve Conduction Studies,” Intensive Care Medicine 40, no. 5 (2014): 683–690, 10.1007/s00134-014-3251-6. 24623137

[15] D. A. Kelmenson , D. Quan , and M. Moss , “What Is the Diagnostic Accuracy of Single Nerve Conduction Studies and Muscle Ultrasound to Identify Critical Illness Polyneuromyopathy: A Prospective Cohort Study. Article,” Critical Care 22, no. 1 (2018): 2281, 10.1186/s13054-018-2281-9. PMC6296115 30558638

[16] P. Formenti , M. Umbrello , V. Castagna , et al., “Respiratory and Peripheral Muscular Ultrasound Characteristics in ICU COVID 19 ARDS Patients,” Journal of Critical Care 67 (2022): 14–20, 10.1016/j.jcrc.2021.09.007. 34600218 PMC8480969

[17] A. Gavazzi , F. de Rino , M. C. Boveri , A. Picozzi , and M. Franceschi , “Prevalence of Peripheral Nervous System Complications After Major Heart Surgery,” Neurological Sciences 37, no. 2 (2016): 205–209, 10.1007/s10072-015-2390-z. 26439918

[18] A. Jain , P. J. Mathew , M. Modi , and K. J. Mangal , “Unilateral Common Peroneal Nerve Palsy Following Renal Transplantation: A Case Report of Tacrolimus Neurotoxicity,” Journal of Postgraduate Medicine 57, no. 2 (2011): 126–128. 21654135 10.4103/0022-3859.81871

[19] A. Ojha , S. A. Zivkovic , and D. Lacomis , “Electrodiagnostic Studies in the Intensive Care Unit: A Comparison Study 2 Decades Later,” Muscle & Nerve 57, no. 5 (2018): 772–776, 10.1002/mus.25998. 29053882

[20] S. Aydın and B. Akın , “Machine Learning Classification of Maladaptive Rumination and Cognitive Distraction in Terms of Frequency Specific Complexity,” Biomedical Signal Processing and Control 77 (2022): 103740, 10.1016/j.bspc.2022.103740.

[21] A. L. Fisse , C. May , J. Motte , et al., “New Approaches to Critical Illness Polyneuromyopathy: High‐Resolution Neuromuscular Ultrasound Characteristics and Cytokine Profiling,” Neurocritical Care 35, no. 1 (2021): 139–152, 10.1007/s12028-020-01148-2. 33236290 PMC7685687

[22] N. A. Ali , J. J. M. O'Brien , S. P. Hoffmann , et al., “(2008) Acquired Weakness, Handgrip Strength, and Mortality in Critically III Patients. Article,” American Journal of Respiratory and Critical Care Medicine 178, no. 3 (2008): 261–268, 10.1164/rccm.200712-1829OC. 18511703

[23] H. D. J. G. Marrero , E. V. Stålberg , G. Cooray , et al., “Neurogenic vs. Myogenic Origin of Acquired Muscle Paralysis in Intensive Care Unit (ICU) Patients: Evaluation of Different Diagnostic Methods,” Diagnostics (Basel) 10, no. 11 (2020): 966, 10.3390/diagnostics10110966. 33217953 PMC7698781

[24] N. Latronico and C. F. Bolton , “Critical Illness Polyneuropathy and Myopathy: A Major Cause of Muscle Weakness and Paralysis,” Lancet Neurology 10, no. 10 (2011): 931–941, 10.1016/S1474-4422(11)70178-8. 21939902

[25] S. Koch , S. Spuler , M. Deja , et al., “Critical Illness Myopathy Is Frequent: Accompanying Neuropathy Protracts ICU Discharge,” Journal of Neurology, Neurosurgery & Psychiatry 82, no. 3 (2011): 287–293, 10.1136/jnnp.2009.192997. 20802220

[26] G. Hermans , B. Clerckx , T. Vanhullebusch , et al., “Interobserver Agreement of Medical Research Council Sum‐Score and Handgrip Strength in the Intensive Care Unit,” Muscle & Nerve 45, no. 1 (2012): 18–25, 10.1002/mus.22219. 22190301

[27] R. Appleton , J. Kinsella , and T. J. J. I. C. S. Quasim , “The Incidence of Intensive Care Unit‐Acquired Weakness Syndromes: A Systematic Review,” Journal of the Intensive Care Society 16, no. 2 (2015): 126–136, 10.1177/1751143714563016. 28979394 PMC5606476

[28] C. F. Bolton , G. Y. Bryan , and D. W. Zochodne , “The Neurological Complications of Sepsis. Review,” Annals of Neurology 33, no. 1 (1993): 94–100, 10.1002/ana.410330115. 8388191

[29] N. Latronico , F. Fenzi , D. Recupero , et al., “Critical Illness Myopathy and Neuropathy,” Lancet 347, no. 9015 (1996): 1579–1582, 10.1016/S0140-6736(96)91074-0. 8667865

[30] R. D. Zorowitz , “ICU–Acquired Weakness,” Chest 150, no. 4 (2016): 966–971, 10.1016/j.chest.2016.06.006. 27312737

[31] T. Sharshar , S. Bastuji‐Garin , R. D. Stevens , et al., “Presence and Severity of Intensive Care Unit‐Acquired Paresis at Time of Awakening Are Associated With Increased Intensive Care Unit and Hospital Mortality,” Critical Care Medicine 37, no. 12 (2009): 3047–3053, 10.1097/CCM.0b013e3181b027e9. 19770751

[32] M. A. C. J. De Letter , P. I. M. Schmitz , L. H. Visser , et al., “Risk Factors for the Development of Polyneuropathy and Myopathy in Critically Ill Patients,” Critical Care Medicine 29, no. 12 (2001): 2281–2286, 10.1097/00003246-200112000-00008. 11801825

[33] S. Nanas , K. Kritikos , E. Angelopoulos , et al., “Predisposing Factors for Critical Illness Polyneuromyopathy in a Multidisciplinary Intensive Care Unit,” Acta Neurologica Scandinavica 118, no. 3 (2008): 175–181, 10.1111/j.1600-0404.2008.00996.x. 18355395

[34] G. Van Den Berghe , K. Schoonheydt , P. Becx , F. Bruyninckx , and P. J. Wouters , “Insulin Therapy Protects the Central and Peripheral Nervous System of Intensive Care Patients,” Neurology 64, no. 8 (2005): 1348–1353, 10.1212/01.WNL.0000158442.08857.FC. 15851721

[35] J. Garnacho‐Montero , J. Madrazo‐Osuna , J. García‐Garmendia , et al., “Critical Illness Polyneuropathy: Risk Factors and Clinical Consequences. A Cohort Study in Septic Patients. Article,” Intensive Care Medicine 27, no. 8 (2001): 1288–1296, 10.1007/s001340101009. 11511941

[36] N. J. Witt , D. W. Zochodne , C. F. Bolton , et al., “Peripheral Nerve Function in Sepsis and Multiple Organ Failure,” Chest 99, no. 1 (1991): 176–184, 10.1378/chest.99.1.176. 1845860

[37] L. Wieske , C. Verhamme , E. Witteveen , et al., “Feasibility and Diagnostic Accuracy of Early Electrophysiological Recordings for ICU‐Acquired Weakness: An Observational Cohort Study,” Neurocritical Care 22, no. 3 (2015): 385–394, 10.1007/s12028-014-0066-9. 25403763

