# Supplementary material for: Rapid Diagnostic Model for Critical Illness Polyneuropathy Based on Electrophysiological Data
Source: CNS Neurosci Ther. 2025 Oct 22;31(10):e70631. doi: 10.1111/cns.70631 (PMC12541361; doi:10.1111/cns.70631)
Supplement: Supplementary file 1 — Data S1: cns70631‐sup‐0001‐Supinfo.docx. [file CNS-31-e70631-s001.docx]

**A new rapid diagnostic model for critical illness polyneuropathy using electrophysiological data**

**The detail of Minimal redundancy maximal relevance (mRMR)**

First, we define the mutual information between two random variables *x* and *y* as

$I(x;y)=\iint p(x,y)\log\frac{p(x,y)}{p(x)p(y)}dxdy$, (1)

where *p*(*x*), *p*(*y*) and *p*(*x,y*) are their individual and joint probability density functions.

In terms of mutual information, the purpose of feature selection is to find a feature set *S* with *m* features$\left\{x_{i}\right\}$, which has the largest relavence with the target class *c*. This scheme, called Max-Relavance, has the following form

$\max D(S,c),$ $D=\frac{1}{|S|}\sum_{x_{i}\in S} I(x_{i};c)$, (2)

where *D* is the mean value of all mutual information values between individual feature *x_i_* and class *c.* It is likely that features selected according to Max-Relevance could have rich redundancy, i.e., the dependency among these features could be large. When two features highly depend on each other, the respective class-discriminative power would not change much if one of them is removed. Therefore, the following minimal redundancy (Min-Redundancy) condition can be added to select mutually exclusive features

$\min R(S),$ $R=\frac{1}{|S|^{2}}\sum_{x_{i},x_{j}\in S} I(x_{i};x_{j})$, (3)

The criterion combining the above two constraints is called “minimal-redundancy-maximal-relevance” (mRMR). We define the operator $\Phi(D,R)$ to combine *D* and *R* and consider the following simplest form to optimize *D* and *R* simultaneously

$\max\Phi(D,R),\Phi=D-R$ (4)

**Table S1** The results of Shapiro-Wilk normality tests for all continuous variables.

|  | CIP Patients | | Controls | |
| --- | --- | --- | --- | --- |
|  | W | P | W | P |
| Age | 0.9321 | p<0.05 | 0.9670 | p<0.05 |
| Creatine Kinase (/l) | 0.9218 | p<0.05 | 0.9540 | p<0.05 |
| Albumin (g/l) | 0.9169 | <0.05 | 0.9099 | <0.05 |
| RBC (/l) | 0.9765 | 0.0681 | 0.9889 | 0.5631 |
| Hb (g/l) | 0.9752 | 0.0542 | 0.9894 | 0.6023 |
| CRP (mg/l) | 0.7071 | <0.05 | 0.9491 | <0.05 |
| Glucose (mmol/l) | 0.9169 | <0.05 | 0.9499 | <0.05 |
| WBC (/l) | 0.6787 | <0.05 | 0.9811 | 0.1518 |
| IL-6 (pg/ml) | 0.7600 | <0.05 | 0.8919 | <0.05 |

**Tabel S2**. The importance ranking of all features.

| Rank | 1 | 2 | 3 | 4 | 5 | 6 | 7 | 8 | 9 | 10 |
| --- | --- | --- | --- | --- | --- | --- | --- | --- | --- | --- |
| Feature | Peroneal nerve(M)-distal CMAP | Interosseous muscle- positive sharp wave | Ulnar nerve(S)- latent period | Tibialis anterior muscle - positive sharp wave | Tibialis nerve(M)-distal latent period | Interosseous muscle - fibrillation | Median nerve(M) -proximal latent period | Quadriceps- fibrillation | Ulnar nerve(M)-distal CMAP | Tibialis anterior muscle - fibrillation |
| Rank | 11 | 12 | 13 | 14 | 15 | 16 | 17 | 18 | 19 | 20 |
| Feature | Peroneal nerve(M)-distal latent period | Biceps brachii - fibrillation | Peroneal nerve(M)-proximal CMAP | Ulnar nerve(M)-proximal latent period | Quadriceps- positive sharp wave | Ulnar nerve(S)- SNAP | Biceps brachii - positive sharp wave | Tibialis nerve-proximal CMAP | Median nerve(S)- latent period | Ulnar nerve(M)-proximal CMAP |
| Rank | 21 | 22 | 23 | 24 | 25 | 26 | 27 | 28 | 29 | 30 |
| Feature | Sural nerve(S)- latent period | Median nerve(M)-proximal CMAP | Peroneal nerve(M)-proximal latent period | Ulnar nerve(M)-distal latent period | Median nerve(S)- SNAP | Peroneal nerve(M)-distal MCV | Median nerve(M)-distal CMAP | Tibialis nerve(M)-distal CMAP | Tibialis nerve(M)-proximal latent period | Median nerve(M)-distal MCV |
| Rank | 31 | 32 | 33 | 34 | 35 | 36 | 37 | 38 | 39 | 40 |
| Feature | Sural nerve(S)-SNAP | Ulnar nerve(S)-SCV | Median nerve(M)-distal latent period | Tibialis nerve(M)-distal MCV | Sural nerve(S)-SCV | Ulnar nerve(M)-distal MCV | Quadriceps- Insertion | Median nerve(S)-SCV | Interosseous muscle- Insertion | Biceps brachii - Insertion |
| Rank | 41 |  |  |  |  |  |  |  |  |  |
| Feature | Tibialis anterior muscle - Insertion |  |  |  |  |  |  |  |  |  |

CMAP: Compound Muscle Action Potential

SNAP: Sensory Nerve Action Potential

MCV: Motor Conduction Velocity

SCV: Sensory Conduction Velocity

M: motor nerve

S: sensory nerve

**Table S3**. Performance of 5-fold cross-validation experiments of different machine learning models with all features on the training set

| Model | SN | SP | Precision | ACC | AUC |
| --- | --- | --- | --- | --- | --- |
| XGBoost | 0.77 | 0.94 | 0.91 | 0.85 | 0.95 |
| SVM_RBF_ | 0.78 | 0.87 | 0.84 | 0.83 | 0.93 |
| SVM_Gauss_ | 0.76 | 0.86 | 0.85 | 0.82 | 0.92 |
| RF | 0.77 | 0.85 | 0.82 | 0.81 | 0.91 |
| KNN | 0.75 | 0.83 | 0.81 | 0.80 | 0.88 |
| NCS (peroneal) | 0.93 | 0.57 | 0.66 | 0.74 | 0.84 |
| NCS (sural) | 0.66 | 0.74 | 0.79 | 0.76 | 0.85 |

**Table S4**. Performance 5-fold cross-validation experiments of different models with 5 features on the training set

| Model | SN | SP | Precision | ACC | AUC |
| --- | --- | --- | --- | --- | --- |
| XGBoost | 0.71 | 0.86 | 0.84 | 0.76 | 0.84 |
| SVM_RBF_ | 0.77 | 0.77 | 0.75 | 0.78 | 0.86 |
| SVM_Gauss_ | 0.74 | 0.76 | 0.73 | 0.75 | 0.83 |
| RF | 0.69 | 0.79 | 0.75 | 0.74 | 0.79 |
| KNN | 0.71 | 0.75 | 0.72 | 0.73 | 0.77 |
| NCS (peroneal) | 0.93 | 0.57 | 0.66 | 0.74 | 0.84 |
| NCS (sural) | 0.66 | 0.74 | 0.79 | 0.76 | 0.85 |

**Table S5**. Performance 5-fold cross-validation experiments of different models with 6 features on the training set

| Model | SN | SP | Precision | ACC | AUC |
| --- | --- | --- | --- | --- | --- |
| XGBoost | 0.72 | 0.86 | 0.85 | 0.79 | 0.88 |
| SVM_RBF_ | 0.70 | 0.90 | 0.86 | 0.80 | 0.90 |
| SVM_Gauss_ | 0.68 | 0.87 | 0.82 | 0.77 | 0.87 |
| RF | 0.69 | 0.81 | 0.76 | 0.75 | 0.85 |
| KNN | 0.71 | 0.79 | 0.75 | 0.73 | 0.81 |
| NCS (peroneal) | 0.93 | 0.57 | 0.66 | 0.74 | 0.84 |
| NCS (sural) | 0.66 | 0.74 | 0.79 | 0.76 | 0.85 |

**Table S6**. Performance 5-fold cross-validation experiments of different models with 7 features on the training set

| Model | SN | SP | Precision | ACC | AUC |
| --- | --- | --- | --- | --- | --- |
| XGBoost | 0.74 | 0.90 | 0.90 | 0.83 | 0.92 |
| SVM_RBF_ | 0.72 | 0.94 | 0.91 | 0.83 | 0.93 |
| SVM_Gauss_ | 0.71 | 0.89 | 0.88 | 0.81 | 0.91 |
| RF | 0.70 | 0.84 | 0.80 | 0.80 | 0.90 |
| KNN | 0.71 | 0.82 | 0.78 | 0.79 | 0.86 |
| NCS (peroneal) | 0.93 | 0.57 | 0.66 | 0.74 | 0.84 |
| NCS (sural) | 0.66 | 0.74 | 0.79 | 0.76 | 0.85 |

**Table S7**. Performance 5-fold cross-validation experiments of different models with 8 features on the training set

| Model | SN | SP | Precision | ACC | AUC |
| --- | --- | --- | --- | --- | --- |
| XGBoost | 0.74 | 0.91 | 0.89 | 0.82 | 0.92 |
| SVM_RBF_ | 0.71 | 0.91 | 0.88 | 0.81 | 0.91 |
| SVM_Gauss_ | 0.71 | 0.88 | 0.85 | 0.79 | 0.88 |
| RF | 0.69 | 0.8 | 0.75 | 0.75 | 0.84 |
| KNN | 0.72 | 0.78 | 0.73 | 0.74 | 0.82 |
| NCS (peroneal) | 0.93 | 0.57 | 0.66 | 0.74 | 0.84 |
| NCS (sural) | 0.66 | 0.74 | 0.79 | 0.76 | 0.85 |

**Table S8**. Performance 5-fold cross-validation experiments of different models with 9 features on the training set

| Model | SN | SP | Precision | ACC | AUC |
| --- | --- | --- | --- | --- | --- |
| XGBoost | 0.73 | 0.92 | 0.89 | 0.81 | 0.91 |
| SVM_RBF_ | 0.71 | 0.90 | 0.87 | 0.81 | 0.91 |
| SVM_Gauss_ | 0.69 | 0.89 | 0.87 | 0.81 | 0.91 |
| RF | 0.7 | 0.81 | 0.76 | 0.77 | 0.83 |
| KNN | 0.71 | 0.90 | 0.74 | 0.75 | 0.79 |
| NCS (peroneal) | 0.93 | 0.57 | 0.66 | 0.74 | 0.84 |
| NCS (sural) | 0.66 | 0.74 | 0.79 | 0.76 | 0.85 |

**Table S9**. Performance 5-fold cross-validation experiments of different models with 10 features on the training set

| Model | SN | SP | Precision | ACC | AUC |
| --- | --- | --- | --- | --- | --- |
| XGBoost | 0.72 | 0.91 | 0.9 | 0.82 | 0.91 |
| SVM_RBF_ | 0.73 | 0.92 | 0.84 | 0.83 | 0.92 |
| SVM_Gauss_ | 0.71 | 0.91 | 0.82 | 0.81 | 0.90 |
| RF | 0.72 | 0.83 | 0.78 | 0.79 | 0.86 |
| KNN | 0.69 | 0.91 | 0.72 | 0.73 | 0.81 |
| NCS (peroneal) | 0.93 | 0.57 | 0.66 | 0.74 | 0.84 |
| NCS (sural) | 0.66 | 0.74 | 0.79 | 0.76 | 0.85 |

**Table S10**. Performance of the rapid diagnostic model and all-feature model on the independent validation set

| Model | SN | SP | Precision | ACC | AUC |
| --- | --- | --- | --- | --- | --- |
| All-feature model | 0.86 | 0.92 | 0.93 | 0.89 | 0.90 |
| Rapid diagnostic model | 0.86 | 0.89 | 0.90 | 0.87 | 0.88 |


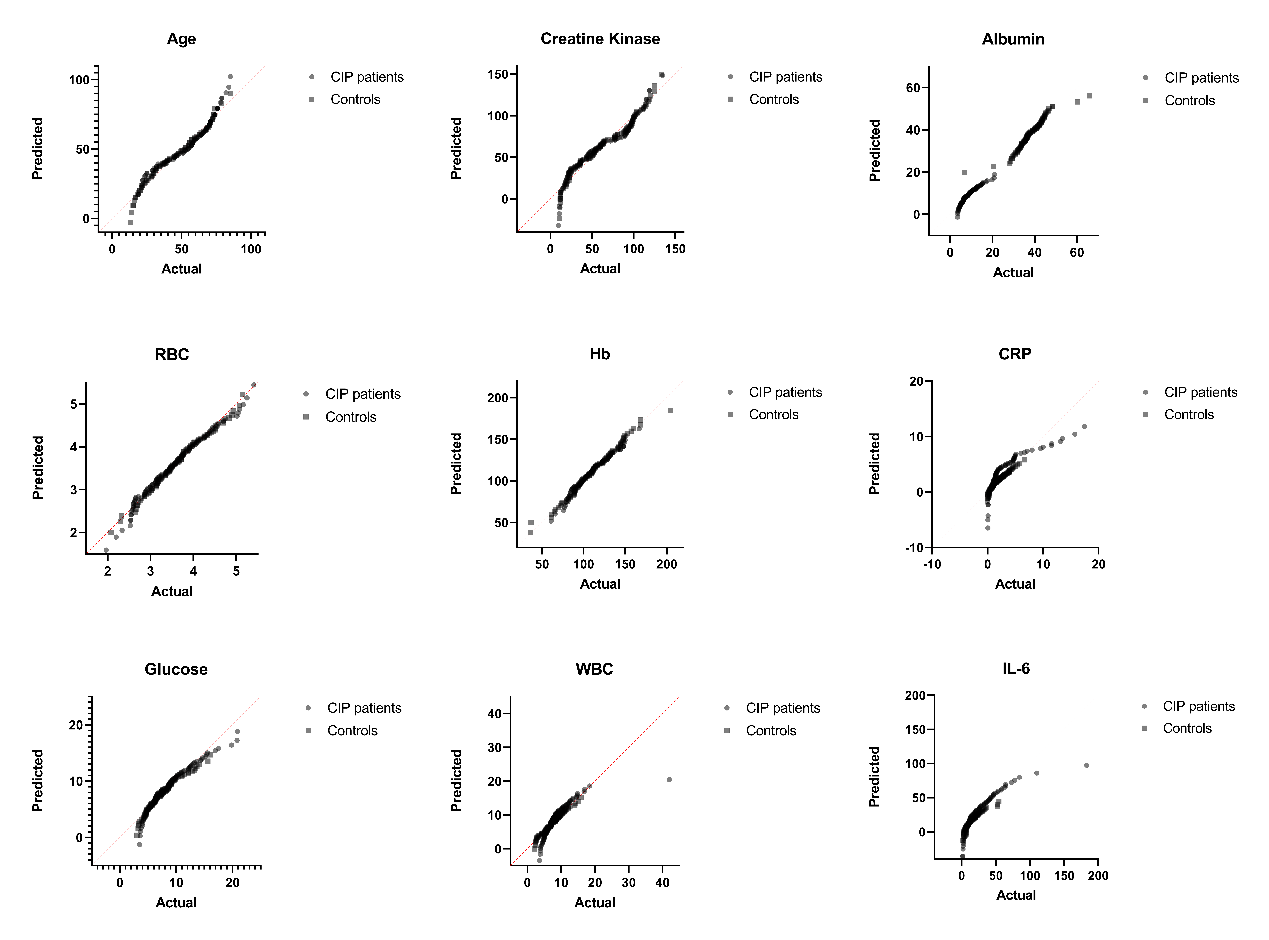


**Figure S1** The Quantile-Quantile Plot for all continuous variables


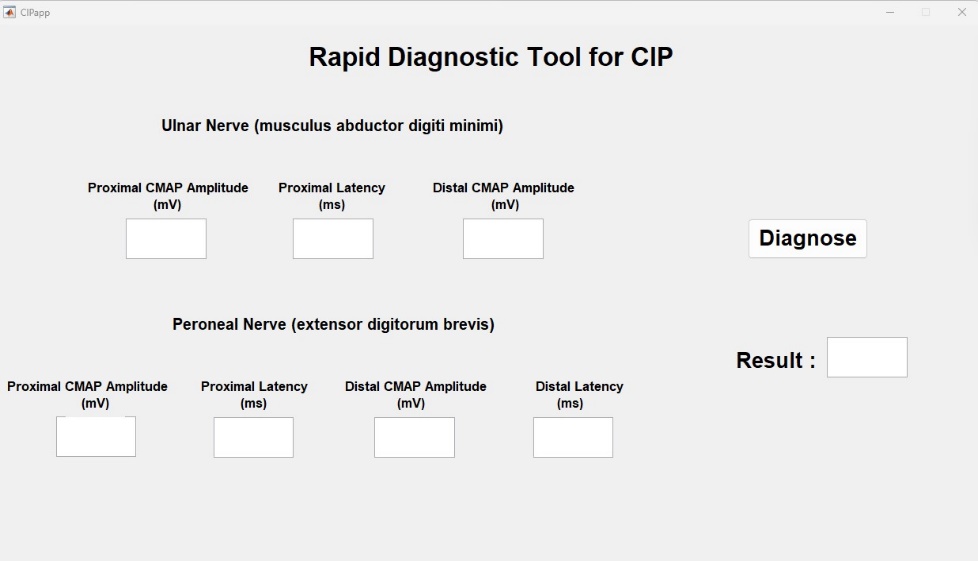


**Figure S2**. The interface of the software


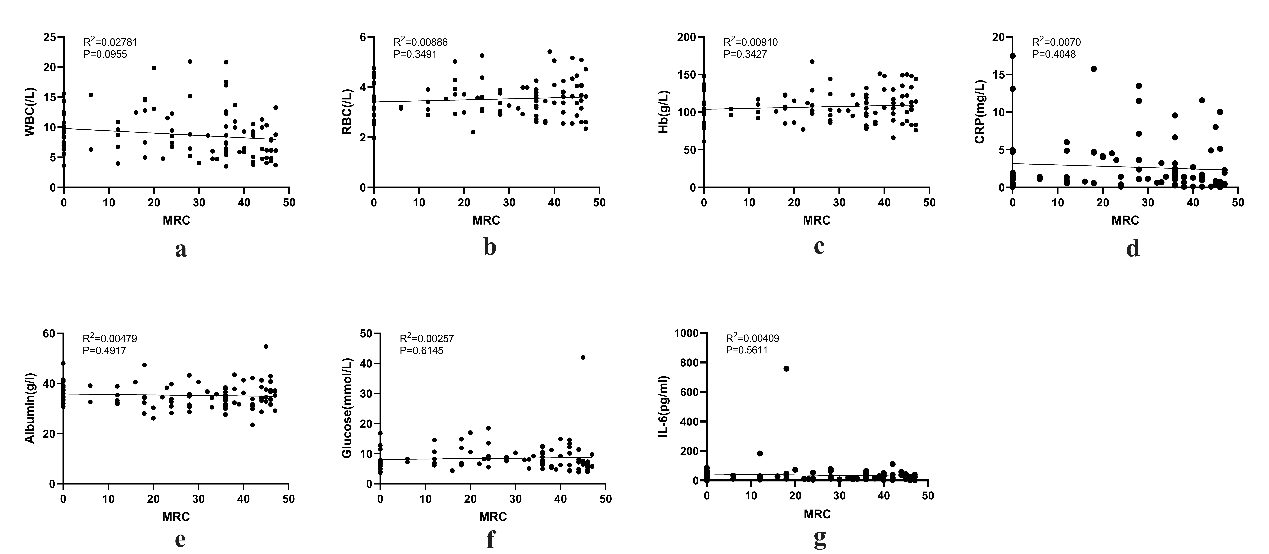


**Figure S3** Correlation of laboratory indicators and MRC in the patients. R2WBC-MRC = 0.02781, P = 0.0955; R2RBC-MRC = 0.00886, P = 0.3491; R2Hb-MRC = 0.0091, P = 0.3427; R2CRP-MRC = 0.007, P = 0.4048; R2Albumin-MRC = 0.00479, P = 0.4917; R2Glucose-MRC = 0.00257, P = 0.6145; R2IL-6-MRC = 0.00409, P = 0.5611

MRC: Medical Research Council;

WBC: White blood cell;

RBC: Red blood cell;

CRP: C-reactive protein;
